# Assessment of *Leishmania* exposure in blood donors from a non‐endemic urban area: A study in São Paulo

**DOI:** 10.1111/vox.70146

**Published:** 2025-11-16

**Authors:** Ismael Severino de Lima, Suzete Cleusa Ferreira, Anna Shoko Nishiya, Norival Kesper, Jerenice Esdras Ferreira, Claudia Maria de Castro Gomes, Juliana Derriga, Katia Cristina Dantas, Silvia Petrossi Gallo Polato, Nanci Alves Salles, Jose Angelo Lauletta Lindoso, Tila Fanciani, Cesar de Almeida‐Neto, Vanderson Rocha, Alfredo Mendrone

**Affiliations:** ^1^ Division of Research & Transfusion Medicine Pro‐Blood Foundation/Blood Center of São Paulo São Paulo Brazil; ^2^ Laboratory of Medical Investigation in Pathogenesis and Targeted Therapy in Onco‐Immuno‐Hematology (LIM‐31), Department of Hematology Hospital das Clínicas, Faculty of Medicine, University of São Paulo São Paulo Brazil; ^3^ Faculdade de Medicina da USP, Laoratorio de Parasitologia Instituto de Medicina Tropical São Paulo Brazil; ^4^ Department of Pathology Instituto Adolf Lutz São Paulo Brazil; ^5^ Department of Pathology, Faculty of Medicine University of São Paulo São Paulo Brazil; ^6^ Department of Medical Sciences, Faculty of Medicine University of São Paulo São Paulo Brazil

**Keywords:** blood donors, *Leishmania*, non‐endemic region, real‐time PCR, seroprevalence, transfusion‐transmitted infection

## Abstract

**Background and Objectives:**

According to the World Health Organization, more than 1 billion people are at risk of leishmaniasis in over 89 countries. Environmental changes such as deforestation, urban expansion and climate change facilitate the spread of sand fly vectors and reservoirs, increasing disease transmission. The introduction of *Leishmania* into non‐endemic regions may be further driven by globalization and ecotourism. Transfusion transmission, particularly of *Leishmania infantum*, remains a concern due to the parasite's ability to survive under blood storage conditions and its prolonged latent phase. We aimed to determine the prevalence of *Leishmania* spp. among blood donors in a non‐endemic region.

**Materials and Methods:**

A prospective, cross‐sectional study was conducted with 5145 blood donor samples collected from January to December 2023. Serological screening was performed using an in‐house immunoglobulin G (IgG) ELISA based on *Leishmania chagasi* antigen. Samples with positive or inconclusive ELISA results were further tested by real‐time PCR targeting internal transcribed spacer (ITS) and kinetoplast DNA (kDNA) regions, according to Pirmez et al.

**Results:**

Among samples tested, 2.82% (141/5145) were ELISA‐reactive. None of these were positive by PCR for ITS or kDNA.

**Conclusion:**

The absence of *Leishmania* DNA in ELISA‐reactive samples highlights the limitations of serological screening in low‐endemicity areas. Inflammatory physiological conditions, such as pregnancy and abortion, may contribute to non‐specific reactivity. The incorporation of molecular methods and the adoption of universal leukoreduction are recommended measures to ensure transfusion safety and avoid unnecessary donor deferrals.

**Highlights**

- The seroprevalence of *Leishmania* among blood donors in a non‐endemic area was 2.82%, but none of the samples were confirmed by PCR.
- The findings suggest that non‐specific ELISA reactivity may be associated with physiological conditions rather than true infection.
- The incorporation of complementary molecular methods and universal leukoreduction is recommended to enhance transfusion safety and avoid unnecessary donor deferrals.

## INTRODUCTION

Leishmaniasis is a neglected tropical disease caused by protozoa of the genus *Leishmania*, transmitted primarily through the bite of infected female phlebotomine sandflies. The disease manifests mainly in two clinical forms: cutaneous and visceral leishmaniasis (VL), the latter being potentially fatal if left untreated. Although vectorial transmission is the most common route, non‐vectorial transmission including congenital, sexual, organ transplantation and blood transfusion has been increasingly recognized, raising significant concerns in transfusion medicine [1].

*Leishmania* parasites have been shown to persist in the peripheral blood of asymptomatic individuals, posing a silent threat to transfusion recipients, particularly immunocompromised patients. While the transfusional transmission of *Leishmania* is not well documented, evidence of parasite DNA in blood donors detected through molecular techniques supports the possibility of this transmission route [1, 2, 3].

In endemic areas, studies have reported substantial prevalence rates of asymptomatic infection among blood donors. For instance, in Mato Grosso do Sul, Brazil, *Leishmania* DNA was detected in 5.4% of healthy donors [1], and 2.8% in Araçatuba, São Paulo, a region with recent VL urbanization [2]. In Teresina, Piauí, another endemic city in Northeastern Brazil, *Leishmania* DNA was found in 3.6% of donors [3]. Similar findings have been observed internationally, with molecular detection rates of 2.8% in blood donors from Iran [4] and 4.2% seroprevalence in India [5].

Interestingly, several studies have also reported *Leishmania* infection in blood donors from non‐endemic regions. In Paraná, Southern Brazil, *Leishmania* DNA was found in 0.4% of donors despite the region being considered non‐endemic [6]. In Madrid, Spain, where VL is not widespread, molecular testing detected *Leishmania infantum* DNA in 0.88% of asymptomatic blood donors [7, 8, 9, 10, 11, 12]. Such findings highlight the potential role of silent carriers and the risk of transfusion‐transmitted leishmaniasis (TTL) even outside recognized endemic zones.

These findings reinforce the importance of active surveillance and the need for targeted screening strategies in endemic regions, particularly for transfusion recipients at high risk, such as immunocompromised individuals. However, a few studies report the prevalence of *Leishmania* spp. in non‐endemic areas. Therefore, the objective of our study was to determine the prevalence of *Leishmania* spp. among blood donors from a non‐endemic region.

## MATERIALS AND METHODS

This was a prospective, cross‐sectional study that included 5145 blood donor samples collected from January to December 2023. Serological screening was performed using an in‐house immunoglobulin G (IgG) ELISA based on *Leishmania chagasi* culture antigen [13]. Samples with positive or inconclusive serological results were further tested by real‐time PCR targeting the internal transcribed spacer (ITS) and kinetoplast DNA (kDNA) regions, following the protocol described by Pirmez et al. as a confirmatory assay [14].

### In‐house ELISA

An in‐house ELISA was performed to detect anti‐*Leishmania* antibodies. Polystyrene microplates (Costar High Binding 3690, Corning, NY, USA) were coated with *L. chagasi* antigen at a concentration of 0.5 μg/mL (50 μL/well) and blocked with 5% skim milk, as previously described [8, 9, 10]. Serum samples were diluted 1:200 and incubated in duplicate at 37°C for 60 min. After five washes with phosphate‐buffered saline (PBS)‐Tween buffer, 50 μL of anti‐human IgG peroxidase‐conjugated antibody (A0170, Sigma‐Aldrich, St. Louis, USA), diluted 1:30,000, was added. Plates were incubated again at 37°C for 60 min followed by additional washing. A chromogenic substrate solution was then added (50 μL/well) and incubated at 37°C for 30 min. The reaction was stopped by adding 1 N hydrochloric acid, and absorbance was read at 492 nm using a microplate reader [13].

### TESA‐blot immunoblotting for *Trypanosoma cruzi*

To exclude *Trypanosoma cruzi* infection, the trypomastigote excreted‐secreted antigen (TESA)‐blot immunoblotting assay was performed following Umezawa et al. [15]. Nitrocellulose membranes containing TESA were blocked with PBS containing 5% skim milk and incubated for 1 h at room temperature (RT) with constant agitation. The membranes were then incubated with serum samples diluted 1:100 in PBS with 1% skim milk for 2 h at RT, followed by five washes with PBS. Next, the membranes were incubated with peroxidase‐conjugated anti‐human IgG (1:4000, Sigma‐Aldrich, USA) for 2 h at RT. After another series of PBS washes, immune complexes were visualized by adding a chromogenic solution consisting of 6 mg of 4‐chloro‐1‐naphthol, 2 mL methanol, 10 mL PBS and 10 μL H_2_O_2_. The reaction was stopped with distilled water upon the appearance of reactive bands, and the membranes were dried between filter papers. A reactive band at 150–160 kDa was considered indicative of *T. cruzi* infection [16].

### Real‐time PCR for *Leishmania*

A highly sensitive in‐house real‐time PCR multiplex assay targeting the ITS ribosomal RNA (rRNA) and kDNA minicircle regions of *Leishmania* spp. was used to quantify parasite load [16, 17, 18]. Total nucleic acids were extracted from 500 μL of whole blood using the MagNA Pure Compact Nucleic Acid Isolation Kit—Large Volume (Roche, Germany), with elution in 100 μL, or alternatively using the QIAamp DNA Blood Mini Kit (Qiagen, Hilden, Germany), according to the manufacturer's instructions. An internal control was added during extraction using 2 μL of poliovirus vaccine (diluted 1:100 in ultrapure water), which was detected by a specific real‐time PCR assay using the following primers and probe: PV_Forward: 5′‐CCCTCCCCTCACAAAAACAG‐3′, PV_Reverse: 5′‐TCACTTGCATGGAGTCTTGCA‐3′ and PV_Probe: 5′‐VIC‐TGGTACTGTTTCCTTGCC‐NFQ‐3′ [17]. For detection of *Leishmania*, amplification was performed using primers L.ITS.F (5′‐CAAATACACGCATGCACTCTC‐3′) and L.ITS.R (5′‐TTTAATAATCCTGGTCACAGCC‐3′), and the probe FAM‐5′‐AGCGTCGAAACTCCTCTCTGGTGC‐3′‐TAMRA. Additionally, primers HM1 (5′‐CCGCCCCTATTTTACACCAACCCC‐3′), HM2 (5′‐GGGGAGGGGCGTTCTGCGAA‐3′) and HM3 (5′‐GGCCCACTATATTACACCAACCCC‐3′) were used to amplify a 120 bp fragment of the conserved kDNA minicircle region of *Leishmania* [16, 18]. Amplification was carried out in a StepOne Plus Real‐Time PCR System (Applied Biosystems, Foster City, CA, USA) with the following cycling conditions: initial denaturation at 95°C for 10 min, followed by 45–50 cycles of 94°C for 15 s and 60°C for 60 s.

### Statistical analysis

This was a prospective cross‐sectional study conducted with 5000 blood donor samples to estimate the prevalence of (disease/agent). Categorical variables were analysed using 2 × 2 contingency tables, with associations assessed by the chi‐square test or Fisher's exact test when expected cell counts were <5. Prevalence was calculated with a 95% confidence interval (CI). A *p*‐value <0.05 was considered statistically significant. All statistical analyses were performed using GraphPad Prism, version 9.5.1 (GraphPad Software, San Diego, CA, USA).

### Statistical estimation of the limit of detection with 95% CI

The limit of detection (LOD) and its 95% CI were estimated using a binary logistic regression model, in accordance with the guidelines provided by the Clinical and Laboratory Standards Institute (CLSI) EP17‐A2 (2012) [19]. Serial dilutions of known target concentrations (expressed in copies/mL) were prepared, and 20 replicate reactions were tested at each concentration level. The proportion of positive results at each dilution was used to model the probability of detection as a function of analyte concentration.

The logistic regression equation applied was:

$$\log\left(1-\mathrm{pp}\right)=\text{β0}+\beta 1\text{X1}+\cdots +\text{βkXk}$$

From the fitted model, the LOD—defined as the concentration at which there is a 95% probability of detection—was determined using the inverse of the logistic function:

$$\mathrm{LOD}=\beta 1\log\left(1-0.950.95\right)-\text{β0}$$

The 95% confidence interval (CI) for the LOD was calculated using the delta method or profile likelihood estimation, as implemented in standard statistical software (e.g., R or Python's statsmodels), based on the standard errors of the estimated regression coefficients.

This method allows for robust estimation of the detection capability and its uncertainty, and is widely accepted for molecular diagnostic validations.

### Ethics statement

Human subjects research was conducted following approval from the Institutional Review Board (CAPPesq) of Hospital das Clínicas, University of São Paulo (online registration CAAE# 62177822.8.0000.0068). All blood donors provided written informed consent before sample collection. For donors aged 16 or 17, signed consent was obtained from a parent or legal representative.

## RESULTS

### Reactivity threshold determination

**FIGURE 1:**
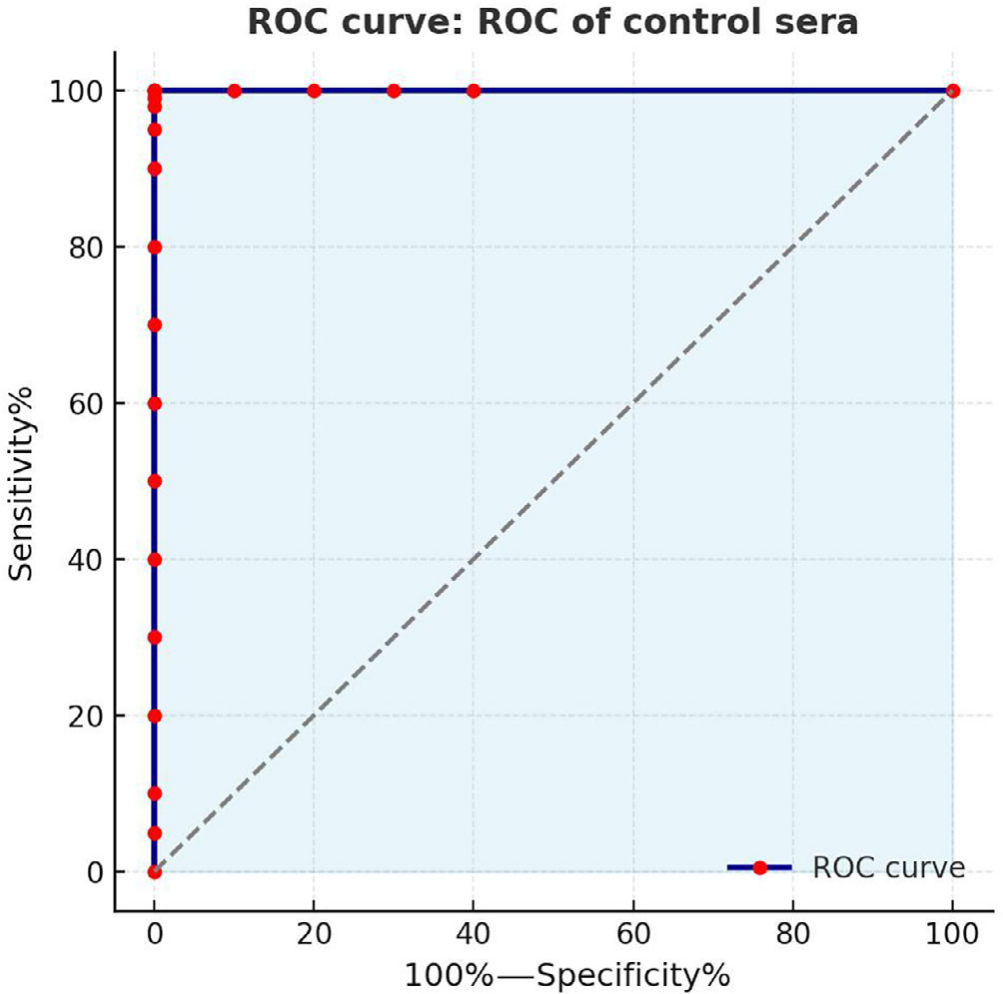
Receiver‐operating characteristic (ROC) curve generated from control sera, showing that the in‐house ELISA for *Leishmania* achieved 100% sensitivity and specificity.

**FIGURE 2:**
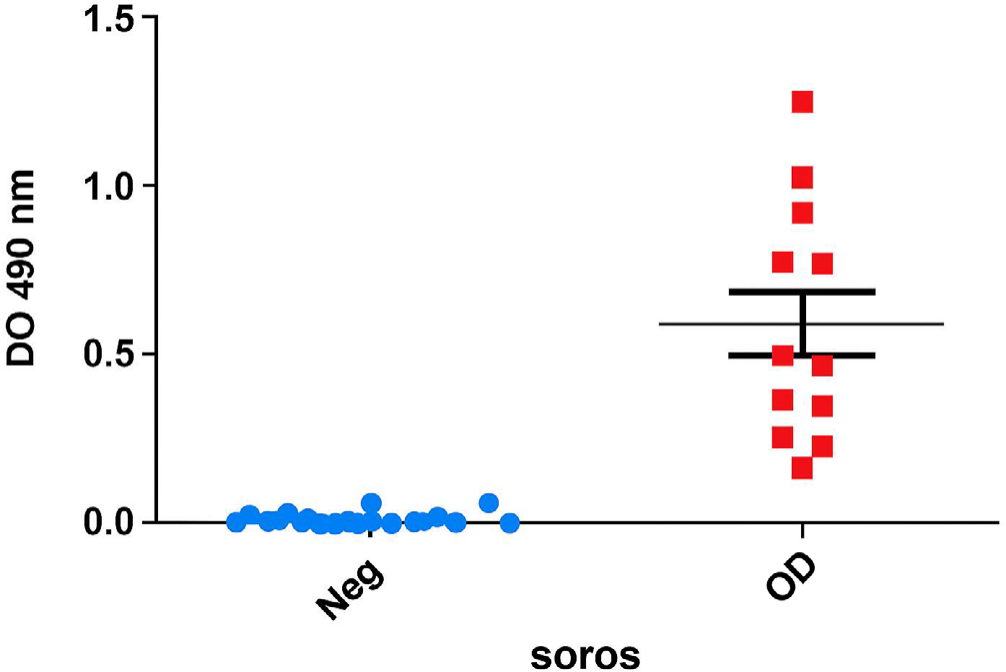
Reactivity results of the panel containing positive and negative controls tested by the in‐house ELISA using *Leishmania chagasi* alkaline extract antigen.

The reactivity threshold (optical density—0.125) of the ELISA was established based on the reactivity of *Leishmania* alkaline extract (EAL) with 22 samples from healthy individuals (*N*) and 12 samples from patients with VL, including cases with high, moderate and low serological reactivity as determined by conventional serology. The cutoff was calculated using the receiver‐operating characteristic (ROC) curve method [19] in Prism™ software version 5.0 (GraphPad Software Inc., 1999) for Windows®. This analysis compared the Abs_492nm_ values of samples from healthy individuals with those from VL patients confirmed by parasitological and/or serological tests (Figure 1). The in‐house ELISA for *Leishmania* spp. demonstrated both 100% sensitivity and specificity (Figures 1 and 2) [19, 20].

Among the 5145 donor samples tested, a seroprevalence of 2.74% (141/5, 145) for *Leishmania* spp. was observed. None of the ELISA‐reactive samples tested positive by real‐time PCR targeting the ITS and kDNA regions.

Sociodemographic data indicated that most reactive individuals were young (under 38 years old), self‐identified as White and had higher education levels; however, none of these variables were statistically significant (*p* > 0.05). Notably, among the 141 ELISA‐positive samples, 31 donors (22.0%) reported having given birth or having experienced a miscarriage, compared to only 6 (0.12%) among ELISA‐negative samples. This association was statistically significant (*p* < 0.0000001; Table 1).

**TABLE 1:** Sociodemographic data of blood donor samples screened using the in‐house *Leishmania chagasi* ELISA.

Variable	*Leishmania* + (*n* = 141)	%	*Leishmania* − (*n* = 5004)	%	*p*‐Value
Age (years)					0.4757
<38	72	51.10	2432	48.60	
39–58	63	44.70	2232	44.60	
>59	6	4.20	340	6.80	
Sex					0.6862
Female	70	49.60	2398	47.90	
Male	71	50.40	2606	52.10	
Education level					0.9783
R1—Never attended school	0	0.00	3	0.06	
R2A—Incomplete elementary	4	2.80	113	2.25	
R3—Complete elementary	7	5.00	221	4.40	
R4—High school	56	39.70	1980	39.60	
R5—Technical course	2	1.40	145	2.90	
R6—Higher education	70	49.70	2475	49.50	
R7—Master's degree	2	1.40	62	1.20	
R8—Doctorate degree	0	0.00	5	0.09	
Race/ethnicity					0.6562
Black	10	7.10	388	7.70	
Mixed (Parda)	34	24.10	1464	29.30	
White	94	66.70	3029	60.50	
Asian (Yellow)	3	2.10	114	2.30	
Indigenous	0	0.00	9	0.20	
Donor type					0.7041
First‐time	48	34.00	1556	31.10	
Repeat	50	35.50	1783	35.60	
Sporadic	43	30.50	1665	33.30	

	*N* = 70	%	*N* = 2404	%	*p*‐Value
Pregnancy or abortion history					<0.0000001
Yes	31	44.30	6	0.25	
No	39	55.70	2398	99.75	

We used the same panel of samples employed to determine the cutoff value of the in‐house ELISA for leishmaniasis to assess whether the *Chagas* Architect test (Abbott) would cross‐react with *Leishmania* spp. All samples that tested positive for *Leishmania* also showed reactivity in the *Chagas* Architect assay.

All samples that initially tested reactive for *Chagas* were excluded from in‐house *Leishmania* ELISA screening, as they could potentially yield false‐positive results due to cross‐reactivity. During the study period, 55 samples that tested positive for *Chagas* by the Architect assay were submitted to confirmatory testing using the TESA‐blot. Among these, 13 (23.6%) were confirmed positive, and 6 (10.9%) showed indeterminate results by TESA‐blot (Table 2).

**TABLE 2:** Results of the trypomastigote excreted‐secreted antigen‐blot immunoblot assay used as a confirmatory test for *Chagas* disease.

TESA‐blot result	*n*	%
Positive	13	23.6%
Negative	36	65.4%
Doubtful	6	10.9%
Total	55	100.0%

Abbreviation: TESA, trypomastigote excreted‐secreted antigen.

### ELISA positivity using epimastigote alkaline extract compared to TESA‐blot

Among the 36 samples (65.4%) that tested negative for *Chagas* disease by the TESA‐blot confirmatory assay, we performed the in‐house *Leishmania* ELISA and real‐time PCR. All results were negative for both tests.

**FIGURE 3:**
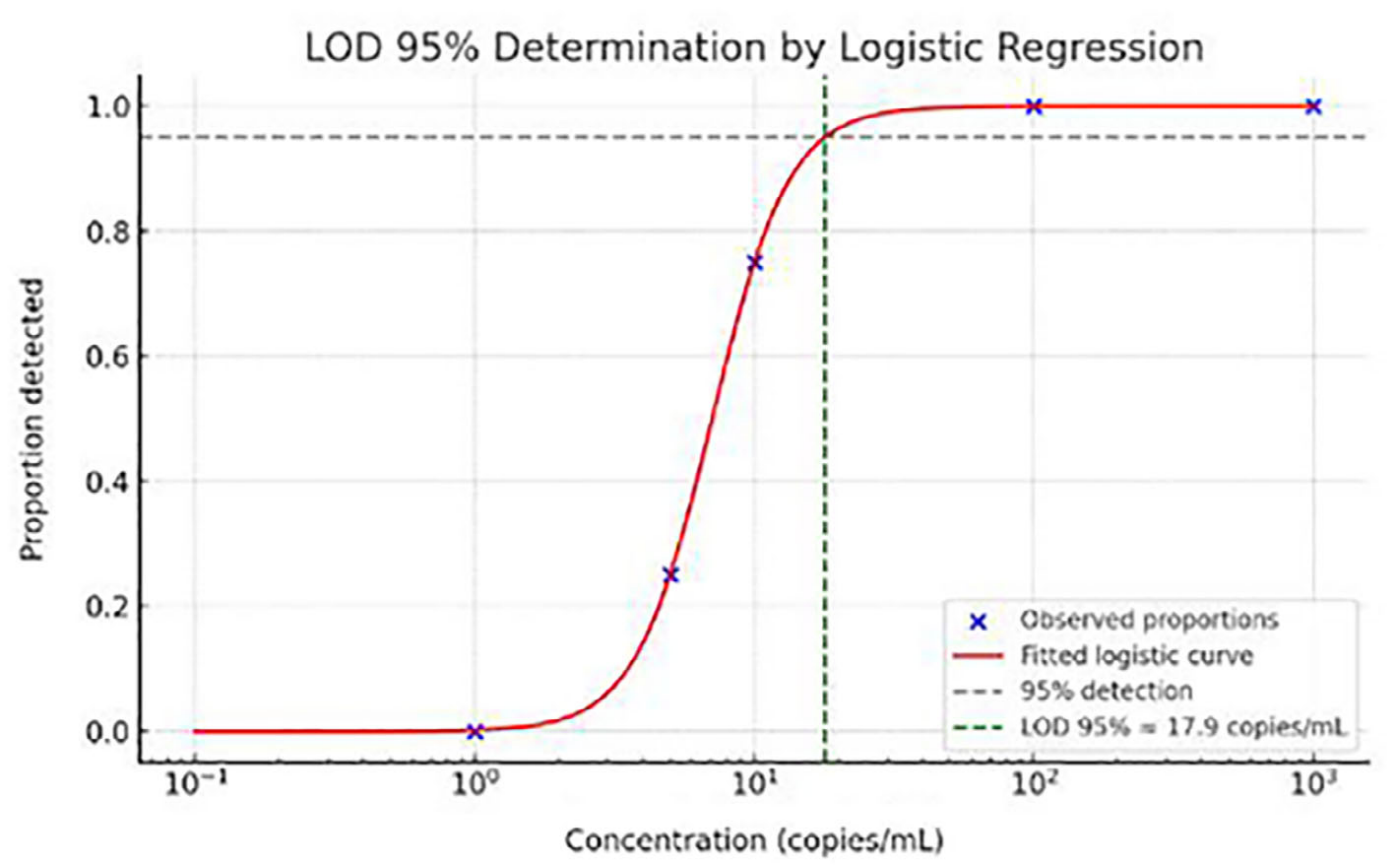
Logistic regression model used to determine the 95% limit of detection (LOD) for the real‐time PCR assay targeting *Leishmania* spp. The curve shows the probability of detection as a function of parasite concentration (copies/mL). The dashed line indicates the 95% detection probability threshold, intersecting the curve at approximately 17.9 copies/mL, representing the assay's LOD with 95% confidence interval.

The LOD at 95% CI for the real‐time PCR assay was estimated at approximately 17.9 copies/mL, based on a logistic regression model. The graph (Figure 3) shows the logistic curve fitted to the experimental data, indicating the intersection point with the 95% detection threshold (Table 3).

**TABLE 3:** Detection of the molecular target at different concentrations.

Concentration (copies/mL)	No. of replicates	No. of positives
1000	20	20
100	20	20
10	20	15
5	20	5
1	20	0
0	20	0

This approach is commonly used to define the analytical sensitivity of molecular assays, as described by Armbruster and Pry [16, 17] and other authors who apply logistic curve modelling to estimate LOD in molecular diagnostics (Table 3 and Figure 3) [16, 17].

**FIGURE 4:**
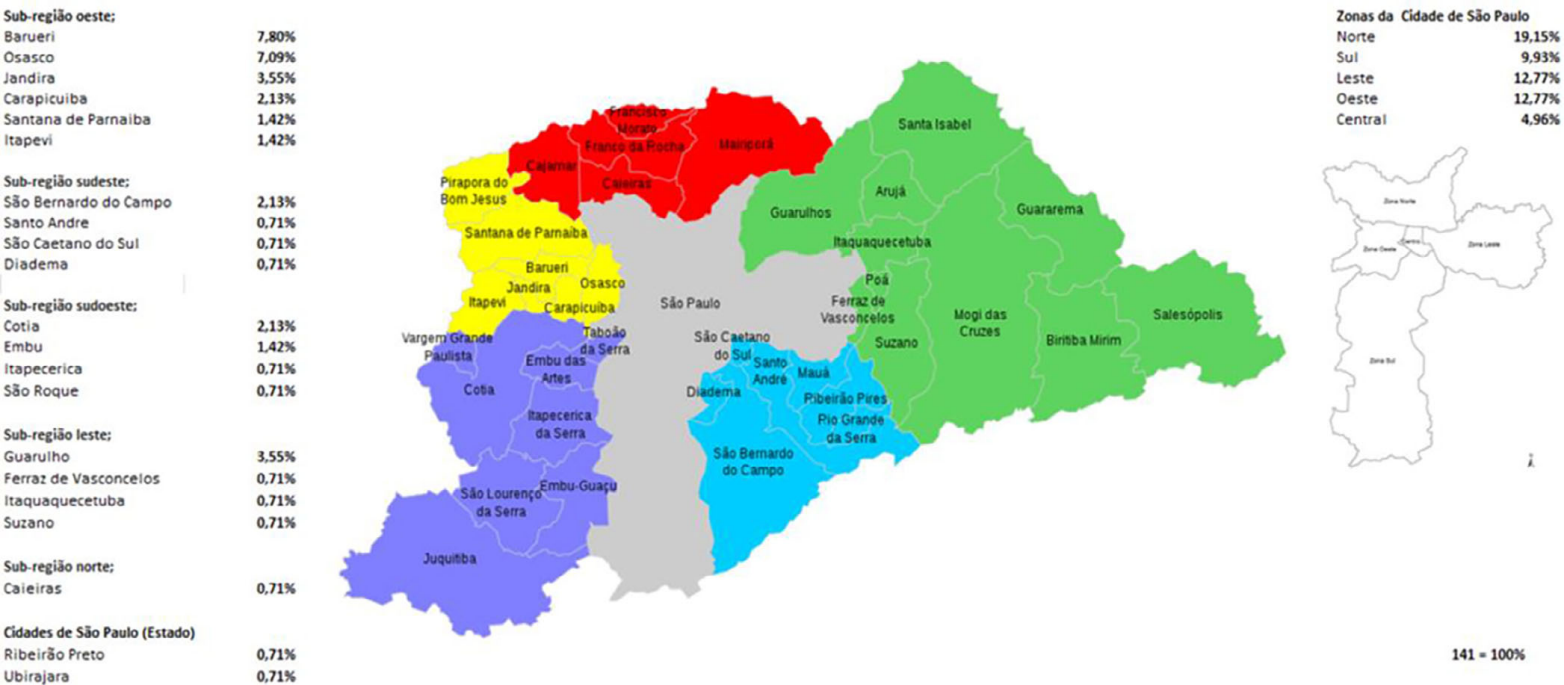
Percentage distribution of the number of cases by region in the State of São Paulo.

When analysing the distribution of seropositive blood donors by residential region, we observed the highest prevalence in the Northern region (19.15%), followed by the Eastern (12.77%), Western (12.77%), Southern (9.93%) and Central (4.96%) regions (Figure 4).

## DISCUSSION

According to the World Health Organization, more than 1 billion people are at risk of contracting leishmaniasis across over 89 countries [21]. Deforestation, unplanned urbanization and the replacement of natural forests with plantations or human dwellings bring people closer to the natural habitats of sand fly vectors and wild reservoirs, thus favouring disease transmission. Other important factors contributing to the spread of leishmaniasis to non‐endemic areas include globalization, climate change and ecotourism [22].

Transfusion‐transmitted *Leishmania* infection has become an increasing concern, particularly in regions endemic for VL, due to the potential for a prolonged latent phase and the parasite's intracellular localization. In this context, universal leukoreduction has been suggested as an effective strategy to reduce transfusion transmission risk, since *Leishmania* primarily resides in leukocytes [23].

In our study, we observed a seroprevalence of 2.74% (141/5145) for *Leishmania* spp. enzyme immunoassay‐reactivity among blood donors from São Paulo, a region considered non‐endemic for VL. However, none of the ELISA‐reactive samples tested positive by real‐time PCR. A comparable investigation carried out in the non‐endemic city of Salvador, Northeastern Brazil, found an anti‐*Leishmania* seroprevalence of 5.4% (38/700) by ELISA, yet none of these seropositive samples demonstrated the presence of parasite DNA by qPCR targeting glucose‐6‐phosphate dehydrogenase, ITS or kDNA regions. This similarity underscores the recurrent issue of serological reactivity without molecular confirmation in low‐endemic settings and reinforces the need for confirmatory testing protocols to ensure transfusion safety and prevent unnecessary donor deferrals [22].

Although ELISA is widely used due to its high sensitivity and ease of implementation, false‐positive results are well‐documented in low‐endemicity settings. In our study, 31 (44.3%) of the 70 women's seroreactive samples were from women who were either pregnant or had a recent history of abortion. Both pregnancy and abortion are associated with systemic inflammatory responses and immune modulation, which can lead to polyclonal B‐cell activation and increased production of non‐specific antibodies, potentially resulting in false‐positive serological results [23]. Moreover, *Leishmania* infection itself has been associated with an increased risk of miscarriage, particularly during active infection, possibly due to parasite‐induced placental inflammation and immunopathology [24].

Screening for *Leishmania* spp. infection in blood donors presents significant challenges, particularly in low‐endemicity settings. In‐house assays, such as ELISA based on alkaline extract antigen from *L. infantum chagasi* (EAL), are frequently used in surveillance studies due to their low cost and reasonable sensitivity [25]. However, the lack of standardization and potential cross‐reactivity with other endemic infections or physiological conditions (e.g., pregnancy) limit their clinical and transfusional applicability [24]. Commercial assays employing recombinant antigens (e.g., rK39 and rK28) offer improved specificity and quality control and are more suitable for systematic screening in blood banks [25]. Nevertheless, their sensitivity may vary depending on the circulating *Leishmania* species, and their higher cost poses a barrier to routine implementation. In the absence of an ideal serological test for blood donor screening, positive results should be interpreted with caution. The use of molecular assays such as PCR as confirmatory tools is recommended [26], along with the adoption of preventive strategies such as universal leukoreduction [27].

The absence of PCR positivity despite ELISA reactivity may reflect low parasite burdens in asymptomatic individuals, residual antibodies from past exposure or non‐specific antibody reactivity in inflammatory conditions. The real‐time PCR assay used in this study had a validated analytical sensitivity of 17.9 copies/mL [26, 27], which is adequate for detecting active infections. Therefore, the lack of detectable *Leishmania* DNA supports the hypothesis that the ELISA‐reactive results do not reflect current parasitaemia.

Sociodemographic analysis revealed higher seroprevalence among young individuals, self‐declared White and with higher educational attainment. This profile differs from that observed in traditionally endemic areas, where leishmaniasis is more common among socioeconomically vulnerable populations. However, the urbanization and expansion of the disease into higher‐income areas, along with better access to health services and routine screening, may explain this pattern in non‐endemic settings [28].

Interestingly, spatial distribution analysis of seroreactive donors revealed a higher prevalence in the Northern region of the state of São Paulo, followed by the Eastern and Western regions. These geographic differences may reflect a combination of ecological, environmental and urban structural factors that influence vector density and human exposure. The Northern region, in particular, is characterized by residual forest fragments, peri‐urban settlements and a higher frequency of canine leishmaniasis, which could contribute to sustained low‐level transmission. Similar patterns were reported by Silva et al. [29], who documented a progressive geographic spread of VL in both human and canine populations in Northern municipalities of São Paulo state. These findings suggest that despite being considered non‐endemic, certain microregions may harbour enzootic transmission cycles, highlighting the need for targeted surveillance and public health interventions [28, 29].

These findings underscore the importance of confirmatory testing, especially in blood donor screening programs in non‐endemic regions. The integration of molecular methods as complementary tools could help reduce unnecessary donor deferrals and enhance transfusion safety when *Leishmania* spp. screen is applied.

Finally, given the potential for subclinical infections, the influence of inflammatory physiological states such as pregnancy and abortion on serological testing, and the limitations of serology in low‐prevalence populations, universal leukoreduction emerges as a feasible and effective measure to reduce the risk of *Leishmania* transmission through transfusion. In parallel, continuous epidemiological surveillance is essential to monitor the potential introduction or re‐emergence of *Leishmania* in urban, non‐endemic areas, especially in light of migration, environmental changes and vector expansion.

In conclusion, in this study, a seroprevalence of 2.74% for *Leishmania* spp. was observed among blood donors from the metropolitan region of São Paulo, an area considered non‐endemic for the disease. However, the absence of PCR positivity in ELISA‐reactive samples suggests that these results do not reflect active infection but possibly residual antibodies or non‐specific reactivity, particularly among pregnant women and those with a history of abortion. These findings highlight the limitations of serological tests in low‐prevalence populations and reinforce the need for the use of confirmatory molecular methods in transfusion screening protocols.

Furthermore, the adoption of complementary strategies, such as universal leukoreduction, is essential to mitigate the risk of transfusion‐transmitted infection, especially in contexts where direct parasite detection is limited. Continuous epidemiological surveillance remains crucial to monitor emerging cases and guide public health policies in response to environmental changes, population mobility and vector expansion into urban areas. These data contribute to the improvement of transfusion safety programs in low‐endemicity regions and emphasize the importance of more specific diagnostic strategies for *Leishmania* spp. in blood banks.

## CONFLICT OF INTEREST STATEMENT

The authors declare no conflicts of interest.

## Data Availability

All data generated or analysed during this study are fully available and presented in the manuscript in the form of tables and figures. No additional datasets were generated or used.
